# Right parietal cortex mediates recognition memory for melodies

**DOI:** 10.1111/ejn.12943

**Published:** 2015-06-08

**Authors:** Nora K. Schaal, Amir‐Homayoun Javadi, Andrea R. Halpern, Bettina Pollok, Michael J. Banissy

**Affiliations:** ^1^ Department of Experimental PsychologyHeinrich‐Heine‐UniversityUniversitätsstraße 140225DüsseldorfGermany; ^2^ Department of Experimental PsychologyInstitute of Behavioural NeuroscienceUniversity College LondonLondonUK; ^3^ Department of PsychologyBucknell UniversityLewisburgPAUSA; ^4^ Medical FacultyInstitute of Clinical Neuroscience and Medical PsychologyHeinrich‐Heine‐UniversityDüsseldorfGermany; ^5^ Department of PsychologyGoldsmithsUniversity of LondonLondonSE14 6NWUK; ^6^ Institute of Cognitive NeuroscienceUniversity College LondonLondonUK

**Keywords:** human, memory for melodies, posterior parietal cortex, recognition, recollection, transcranial direct current stimulation

## Abstract

Functional brain imaging studies have highlighted the significance of right‐lateralized temporal, frontal and parietal brain areas for memory for melodies. The present study investigated the involvement of bilateral posterior parietal cortices (PPCs) for the recognition memory of melodies using transcranial direct current stimulation (tDCS). Participants performed a recognition task before and after tDCS. The task included an encoding phase (12 melodies), a retention period, as well as a recognition phase (24 melodies). Experiment 1 revealed that anodal tDCS over the right PPC led to a deterioration of overall memory performance compared with sham. Experiment 2 confirmed the results of Experiment 1 and further showed that anodal tDCS over the left PPC did not show a modulatory effect on memory task performance, indicating a right lateralization for musical memory. Furthermore, both experiments revealed that the decline in memory for melodies can be traced back to an interference of anodal stimulation on the recollection process (remember judgements) rather than to familiarity judgements. Taken together, this study revealed a causal involvement of the right PPC for memory for melodies and demonstrated a key role for this brain region in the recollection process of the memory task.

## Introduction

An important factor for the enjoyment and communicative role of music is the ability to encode (and later recognise) tunes. The neural structures of auditory memory, especially for melodies, are not well known, and the aim of the present study was to investigate the causal involvement of the right and left posterior parietal cortices (PPCs) in recognition memory for melodies, using transcranial direct current stimulation (tDCS).

Behavioural research on memory for melodies has looked at the influence of surface parameters, such as timbre or tempo, on musical memory (Halpern & Müllensiefen, 2008; Lange & Czernochowski, 2013) but only a few studies have investigated its neural correlates. A recent study using magnetoencephalography showed increased activation of the superior temporal gyri as well as the right inferior temporal gyrus, inferior frontal gyrus and parietal areas for auditory short‐term memory (Nolden *et al*., 2013). Using positron emission tomography, Platel *et al*. (2002) revealed activation of bilateral middle frontal areas (Brodmann areas 9 and 10) and bilateral (but predominantly right‐sided) precuneus (Brodmann area 7) for episodic memory for short melodies. Furthermore, Klostermann *et al*. (2009) showed the activation of the right PPC for the retrieval of short tunes using functional magnetic resonance imaging.

Recognition memory is not a unitary construct. According to the dual‐process model (Yonelinas, 2002), remember/know responses index two types of recognition responses: true recollection ‘I really remember that item’ vs. familiarity ‘that item seems familiar’. These responses also characterise musical memory (Gardiner *et al*., 1996; Lange & Czernochowski, 2013). Brain imaging studies support a distinction between recollection and familiarity (Ranganath *et al*., 2004; Yonelinas *et al*., 2005). Yonelinas *et al*. (2005) revealed that recollection was associated with increased activation of the lateral parietal cortex and posterior cingulate, whereas familiarity judgements activated the superior lateral parietal cortex and precuneus. To our knowledge, brain areas causally involved in musical remember/know responses have not yet been studied.

Transcranial direct current stimulation is a useful tool to investigate the causal involvement of targeted brain areas in cognitive tasks (Nitsche & Paulus, 2001). Research in the motor domain typically links anodal tDCS to a facilitation of neural activity, whereas cathodal tDCS more likely suppresses the cortical excitability under the site of stimulation (Nitsche & Paulus, 2000). Previous tDCS studies on pitch memory have revealed a causal link between the left supramarginal gyrus and pitch recognition and recall (Vines *et al*., 2006; Schaal *et al*., 2013, 2014b), as well as between Heschl's gyrus and pitch discrimination (Mathys *et al*., 2010). Thus, we examined the causal involvement of the bilateral PPC for memory for whole melodies. As Klostermann *et al*. (2009) showed activation in the right PPC during auditory non‐verbal memory, we first examined the right PPC in Experiment 1 by applying either anodal or sham stimulation. In Experiment 2 we compared the effect of anodal tDCS over the right and left PPC. Furthermore, the study investigated whether recollection and familiarity judgements in memory for tunes can be dissociated at the neural level.

## Materials and methods

### Participants

A total of 27 participants with a mean age of 23.8 years (SD 5.1, range 19–41) took part in Experiment 1. They were randomly assigned to the anodal (*N* = 13; six males, seven females; mean age 25.0 years, SD 6.5, range 19–41) or sham (*N* = 14; five males, nine females; mean age 22.7 years, SD 3.0, range 19–30) condition. One female participant from the sham group was distracted by the stimulation and was excluded from the analysis. Twenty‐four novel participants (who did not participate in Experiment 1) with a mean age of 24.8 years (SD 4.1, range 21–40) took part in Experiment 2. They were assigned to one of the two groups: anodal tDCS over the right PPC (*N* = 12; four males, eight females; mean age 23.5 years, SD 2.7, range 21–29) or anodal tDCS over the left PPC (*N* = 12; four males, eight females; mean age 26.1 years, SD 4.8, range 21–40). Groups were matched by baseline memory performance (see Procedure section). Table 1 summarises the demographic details of the final sample. All participants were right‐handed non‐musicians (< 2 years of formal musical training in the past, not playing an instrument at present). They signed a written informed consent and received either course credits or 8 Euros for their participation. The study was approved by the local research ethics committee at Heinrich‐Heine‐University in accordance with the Declaration of Helsinki.

**Table 1:** Characteristics and matched baseline performance for the groups of both experiments

	Experiment 1		Experiment 2	
	Anodal right PPC	Sham right PPC	Anodal right PPC	Anodal left PPC
*N*	13	13	12	12
Gender	6 males/7 females	5 males/8 females	4 males/8 females	4 males/8 females
Age (years)	25.00 (6.54)	22.15 (2.30)	23.50 (2.71)	26.08 (4.79)
Musical training (Gold‐MSI score)	11.62 (3.89)	11.77 (3.52)	10.58 (2.71)	13.31 (5.14)
Baseline performance (dʹ)	1.45 (0.81)	1.30 (0.67)	1.19 (0.44)	1.17 (0.54)

SDs are given in parenthesis. Gold‐MSI, Goldsmiths Musical Sophistication Index.

### Material

#### Questionnaire

The German version of the Goldsmiths Musical Sophistication Index questionnaire (Müllensiefen *et al*., 2014; Schaal *et al*., 2015a) was used to evaluate musical sophistication and engagement. The scale consists of 31 statements, which are rated on a seven‐point Likert scale, as well as another seven items asking for the amount of time spent on musical activities. The relevant Musical Training dimension consists of seven statements and a score that ranges between 7 and 49. The low mean score of 11.9 points of the present sample confirms that non‐musicians took part.

### Acoustic stimuli

**Figure 1:**
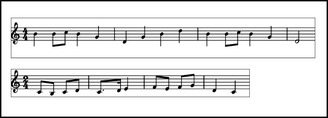
Two examples from the 48 melodies of the memory recognition task.

The 48 stimuli were short, unfamiliar tonal melodies that were derived from folk songs or that were unrecognisable permutations of familiar tunes. The average duration was 6.2 s (range 4–10 s) and they were single‐line melodies synthesised in a MIDI piano timbre (two examples in Fig. 1). They were split into four blocks (A–D) of 12 melodies, respectively. Blocks A and B, as well as C and D, comprised a set of old and new melodies. Sets, as well as the block order within sets, were counterbalanced among participants. Pilot testing ensured that the sets were of the same difficulty.

### Transcranial direct current stimulation

In Experiment 1, the anode electrode (5 × 5 cm^2^) was placed over the right PPC and the reference electrode (5 × 7 cm^2^) was located over the left supraorbital area. An electroencephalographic electrode cap was used to mark the position of the right PPC at P4 (according to the international 10–20 system) as per previous studies (e.g. Bardi *et al*., 2013). In Experiment 2, the anode electrode was either placed over the right PPC (P4) or the left PPC (P3). The reference electrode was placed over the contralateral supraorbital area. A reference electrode that was slightly larger compared with the anode electrode was used to achieve a more focal stimulation (Nitsche *et al*., 2007). The electrodes were covered in saline‐soaked sponges and were fixed on their positions with self‐adhesive bandages. Participants in the anodal stimulation conditions received 15 min of 2 mA direct current stimulation (with 15 s fade in and fade out, respectively), whereas the sham group received the same fade in and fade out, but did not receive any active current during the 15 min stimulation window. Sham stimulation evoked the somatosensory sensation of being stimulated, but did not lead to a neurophysiological change that can influence performance. Participants were not informed about which stimulation they received.

### Procedure

**Figure 2:**
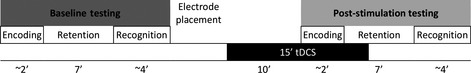
Procedure of the experiment. An experimental session consisted of two phases of baseline and post‐stimulation testing. Each phase consisted of three parts of encoding, retention and recognition. Participants were asked to memorise a set of 12 melodies during the encoding parts. Subsequently, they were presented with 24 melodies of which 12 were old (previously presented in the encoding phase) and 12 were new. They were asked to indicate whether the melody was old or new and, if old, whether they remember it or know it. Numbers indicate the durations in minutes.

Both experiments consisted of three parts and they were identical in their set‐up: baseline testing of memory, tDCS stimulation and post‐manipulation testing of memory (see Fig. 2).

For baseline testing, the participants listened to 12 tunes, each played once, via headphones with 3 s between each tune in the exposure phase. They were asked to memorise the melodies. During the retention phase, the participants worked on a word puzzle for 7 min to prevent rehearsal of the melodies. In the recognition phase, the participants listened to a set of 24 melodies (comprising the 12 melodies from the encoding phase and 12 new melodies). They were required to indicate whether the presented melody was new or old. If they classified a melody as old, they were asked to specify a *remember* or *know* judgement. The instruction stated that *remember* meant that one was consciously aware that this melody was presented before, or true recollection. A *know* response was explained as a feeling of having heard the melody in the encoding phase without any conscious memory of having heard it before, indicating familiarity.

After the baseline testing, electrodes were placed on the scalp. The stimulation lasted for 15 min. The participants were asked to relax, within the first 10 min of the stimulation, then the post‐stimulation testing started, consisting of the exposure phase (with 12 new melodies), the word puzzle, and the test phase (24 melodies). It has been shown that the after‐effects of 11 min of 1 mA tDCS over the motor cortex last up to 1 h, as measured by motor‐evoked potentials (Nitsche & Paulus, 2001). Therefore, we expected to modulate the whole memory process (encoding and retrieval) in the anodal conditions. After completing the tasks, the electrodes were removed and the participants filled in the German version of the Goldsmiths Musical Sophistication Index questionnaire.

### Statistical analysis

For the explicit memory for melodies, we computed dʹ scores (Green & Swets, 1966) both overall, and separately for the ‘remember’ (recollection) and ‘know’ (familiarity) responses (R‐d and K‐d).

For overall memory performance a mixed‐factor anova was conducted for each experiment with the within‐subject factor phase (pre‐stimulation vs. post‐stimulation) and between‐subject factor stimulation condition (anodal vs. sham stimulation in Experiment 1 and anodal right PPC vs. anodal left PPC in Experiment 2) on overall dʹ scores. Cohen's d effect sizes with confidence intervals (CIs) are reported as a measure of effect size. If applicable, *post‐hoc t*‐tests were conducted to disentangle significant interactions. Bonferroni corrections were applied appropriately. Furthermore, performance differences were calculated by subtracting baseline dʹ scores from the dʹ scores after stimulation, reflecting the memory performance change due to stimulation. Independent‐sample *t*‐tests were conducted to check for the influence of stimulation between the two groups of each experiment.

Additionally, mixed‐factor anovas were also conducted for recollection on R‐dʹ and for familiarity on K‐dʹ with the identical within‐subject and between‐subject factors for both experiments.

## Results

Table 2 gives an overview of mean performances of the groups of Experiment 1 and Experiment 2. Overall performance on the memory for melody task was above chance (dʹ ≥ 0) for every individual participant.

**Table 2:** Overview of group performances on overall memory performance as well as ‘remember’ and ‘know’ scores for both experiments

	Experiment 1		Experiment 2	
	Anodal right PPC	Sham right PPC	Anodal right PPC	Anodal left PPC
Baseline overall dʹ	1.45 (0.81)	1.30 (0.67)	1.19 (0.44)	1.17 (0.54)
Stimulation overall dʹ	**0.79 (0.46)**	1.39 (0.62)	**0.79 (0.55)**	1.42 (0.79)
Baseline remember dʹ	1.58 (0.87)	1.33 (1.06)	1.36 (0.55)	1.22 (0.63)
Stimulation remember dʹ	**0.44 (1.1)**	1.22 (0.68)	**0.66 (0.88)**	1.07 (0.63)
Baseline know dʹ	0.41 (0.96)	0.54 (0.80)	0.37 (0.58)	0.46 (0.68)
Stimulation know dʹ	0.52 (0.60)	0.61 (0.49)	0.39 (0.57)	0.62 (0.42)

SDs are given in parenthesis. The bold values highlight the group performances that show a significant modulation effect.

**Figure 3:**
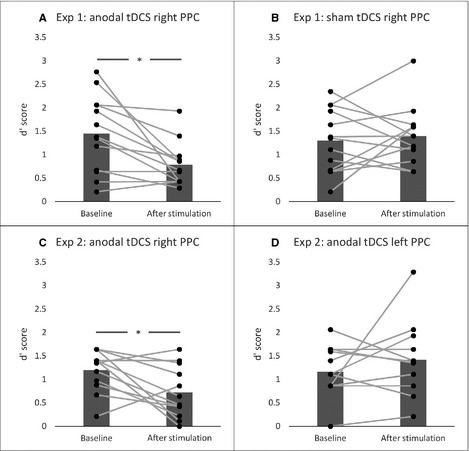
Summary of overall dʹ scores for different conditions and experiments including individual data points to show individual differences. (A) In Experiment 1, anodal tDCS over the right PPC led to a significant decline of overall performance on memory for melodies (*t*_12_ = 3.31, *P *=* *0.006). (B) Sham stimulation over the right PPC showed no modulation effect. (C) Experiment 2 confirmed the significant deterioration of performance after anodal tDCS over the right PPC (*t*_11_ = 3.31, *P *=* *0.03). (D) No significant modulation effect could be found when stimulation was applied over the left PPC. *P < 0.05.

**Figure 4:**
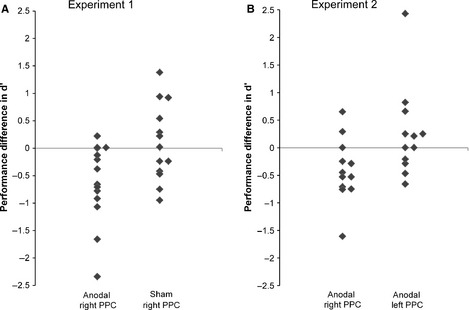
(A, B) The scatterplots show performance differences (post‐stimulation performance – baseline performance) for individual participants. Negative values represent a decline in performance compared with baseline, whereas positive values represent an improvement. In both experiments the decline in performance in the group receiving anodal tDCS over the right PPC was significantly different to the control groups (*P *<* *0.05).

For Experiment 1, a mixed‐factor anova with the within‐subject factor *phase* (pre‐stimulation vs. post‐stimulation) and between‐subject factor *stimulation condition* (anodal vs. sham stimulation) on overall dʹ scores revealed a borderline significant effect of *phase* [*F*_1,24_ = 4.13, *P *=* *0.053, 95% CI (0.00, 1.60), d = 0.79], a non‐significant result for *stimulation condition* [*F*_1,24_ = 1.16, *P *=* *0.292, 95% CI (−0.35, 1.20), d = 0.42] and a significant *phase* × *stimulation condition* interaction [*F*_1,24_ = 7.36, *P *=* *0.012, 95% CI (0.24, 1.89), d = 1.06]. *Post‐hoc* paired‐samples *t*‐tests revealed a significant decline of performance in the anodal group after stimulation (*t*_12_ = 3.31, *P *=* *0.006) and no significant difference pre‐stimulation and post‐stimulation in the sham group (*t*_12_ = 0.19, *P *=* *0.633) (Fig. 3A and B). Additionally, an independent‐samples *t*‐test was conducted on performance differences and *group* as the between‐subject variable, which showed a significant difference [*t*_24_ = 2.71, *P *=* *0.012, 95% CI (0.25, 1.97), d = 1.11]. The reduction of memory performance in the group receiving anodal tDCS over the right PPC (M = −0.66, SD 0.72) was significantly different from the performance difference in the sham group (M = 0.09, SD 0.70) (Fig. 4).

**Figure 5:**
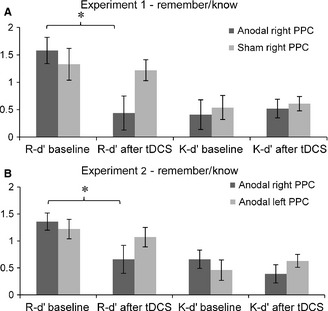
Summary of dʹ scores for recollection (remember) and familiarity (know) in both experiments. (A) In Experiment 1, the analysis on remember and know dʹ scores reveals a significant modulation effect of tDCS over the right PPC on remember judgements (*t*_12_ = 6.48, *P *<* *0.001). (B) Experiment 2 confirms the interference of anodal tDCS over the right PPC on remember responses (*t*_11_ = 2.6, *P *=* *0.025). No effects were found on know responses. The error bars indicate the SEM. *P < 0.05.

For recollection, a mixed‐factor anova with the factors *phase* and *stimulation condition* and the dependent variable R‐dʹ revealed a non‐significant result for *stimulation condition* [*F*_1,24_ = 0.69, *P *=* *0.415, 95% CI (−0.45, 1.10), d = 0.33], a significant effect of *phase* [*F*_1,24_ = 10.85, *P *=* *0.003, 95% CI (0.45, 2.14), d = 1.29], as well as the *phase* × *stimulation condition* interaction [*F*_1,24_ = 7.17, *P *=* *0.013, 95% CI (0.23, 1.87), d = 1.05]. *Post‐hoc* paired‐samples *t*‐tests showed a significant decline after anodal stimulation (*t*_12_ = 5.48, *P *<* *0.001) and no significant difference pre‐stimulation and post‐stimulation in the sham group (*t*_12_ = 0.37, *P *=* *0.72) (Fig. 5A). A similar mixed‐factor anova for familiarity showed no significant effects (*P*‐values > 0.538).

For Experiment 2, a mixed‐factor anova with *phase* (pre‐stimulation vs. post‐stimulation) and *stimulation condition* (anodal right PPC vs. left PPC) on overall dʹ scores revealed non‐significant results for the factors *phase* [*F*_1,22_ = 0.32, *P *=* *0.576, 95% CI (−0.57, 1.03), d = 0.23] and *stimulation condition* [*F*_1,22_ = 2.34, *P *=* *0.141, 95% CI (−0.19, 1.44), d = 0.79], but a significant *phase* × *stimulation condition* interaction [*F*_1,22_ = 5.32, *P *=* *0.031, 95% CI (0.10, 1.79), d = 0.94]. *Post‐hoc* paired‐samples *t*‐tests confirmed the significant decline of anodal tDCS in the group receiving anodal stimulation over the right PPC (*t*_11_ = 3.31, *P *=* *0.03), thus replicating the findings from Experiment 1. No significant difference was found in the group receiving anodal stimulation over the left PPC (*t*_11_ = 0.19, *P *=* *0.310) (Fig. 3C and D). Additionally, the independent‐samples *t*‐tests on performance differences showed that the decline of the anodal tDCS right PPC group (M = −0.41, SD 0.57) was significantly different from the performance change of the anodal tDCS left PPC group [M = 0.25, SD 0.81) (t_22_ = 2.31, *P *=* *0.031, 95% CI (0.1, 1.87), d = 0.99] (Fig. 4B).

In addition, we conducted planned paired‐samples comparisons on R‐dʹ to analyse the recollection memory performance (Wilcox, 1987). A significant decline after anodal stimulation over the right PPC (*t*_11_ = 2.6, *P *=* *0.025) and no significant difference pre‐stimulation and post‐stimulation in the group receiving anodal tDCS over the left PPC (*t*_11_ = 0.51, *P *=* *0.621) was revealed (Fig. 5B). The familiarity analysis on K‐dʹ revealed no significant modulation effects (*P*‐values > 0.380) (Fig. 5B).

## Discussion

The results of Experiment 1 show that anodal tDCS over the right PPC, compared with sham stimulation, modulates performance and highlights a pivotal role for the right PPC in recognition memory for melodies. Anodal tDCS over the right PPC led to a decline in overall memory performance. Additionally, the analyses of recollection and familiarity showed that anodal tDCS over the right PPC led to deterioration in conscious recollection judgements only. Experiment 2 was then conducted to replicate the decline in memory seen in the first experiment. Also, because functional magnetic resonance imaging studies report a strong rightward activation for musical and auditory non‐verbal material (Zatorre *et al*., 1994, 2002; Wagner *et al*., 1998), whereas verbal memory processes are usually referred to left‐hemispheric involvement (Wagner *et al*., 1998; D'Arcy *et al*., 2004; Javadi & Walsh, 2012), we compared right and left PPC active stimulation. Experiment 2 confirms the decline of memory for melodies after anodal tDCS over the right PPC and shows a hemispherically specific modulation of melody as anodal tDCS over the left PPC had no effect. Furthermore, Experiment 2 also shows that the decline in performance was isolated to interference with the *remember* judgements, suggesting the independence of recollection and familiarity during recognition.

Our results are in accordance with brain imaging studies that have highlighted the activation of the right parietal cortex during recognition memory for musical material (Platel *et al*., 2002; Klostermann *et al*., 2009). This is the first study to show a causal involvement of the right (but not left) PPC for memory for melodies, which confirms previous studies claiming a dominant activation of a right‐hemispheric neural circuit for non‐verbal musical memory (Wagner *et al*., 1998; Platel *et al*., 2002; Nolden *et al*., 2013).

Additionally, the results contribute to the theory that recollection and familiarity are independent components of the memory process (Ranganath *et al*., 2004; Yonelinas *et al*., 2005; Vilberg & Rugg, 2008; Evans & Wilding, 2012) as modulation effects were only found on *remember* judgements. This highlights the involvement of the right PPC for recollection. There are at least three reasons that may account for the lack of modulation on familiarity judgements: (i) the right PPC is selectively involved in recollection of recognition memory; (ii) this process is less sensitive to modulation effects given the automatic nature of familiarity memory processes; and (iii) the non‐significant result is due to a floor effect (performance of *know* responses was relatively low overall). It will be important to disentangle these possibilities with additional research.

Interestingly, anodal stimulation of the right PPC led to a drop in recognition memory performance rather than the predicted increase in Experiment 1. Even though the majority of tDCS studies link anodal tDCS to a facilitation of cognitive performances (e.g. Ladeira *et al*., 2011; Javadi & Walsh, 2012; Santiesteban *et al*., 2012; Schaal *et al*., 2013), several studies have also reported deterioration of performance after anodal stimulation (Ferrucci *et al*., 2008; Jones & Berryhill, 2012; Kaminski *et al*., 2013). A recent tDCS study on an auditory between‐channel gap detection task showed a significant decline in performance after anodal stimulation over the left auditory cortex (Heimrath *et al*., 2014). Three possible reasons for the deleterious effect of stimulation on musical memory are as follows.

1. Anodal tDCS over the right PPC may have secondary effects on brain regions being functionally connected with the PPC, such as the dorsolateral prefrontal cortex and medial temporal lobe (Shimamura, 2011), thus influencing memory performance (Chib *et al*., 2013; Notturno *et al*., 2014).
2. The relationship between ratios of excitation and inhibition in the right PPC and cognitive performance may be important. Krause *et al*. (2013) suggested an inverted U‐shape for the influence of anodal stimulation driven by the balance of excitation and inhibition of gamma‐aminobutyric acid and glutamate levels. This result implies that anodal stimulation can improve cognitive performance to a certain point where optimum performance is reached, but if stimulation evokes too much excitability, then a reduction in performance may occur.
3. The signal‐to‐noise ratio may also play a pivotal role in the interpretation of the results. It might be the case that anodal tDCS over the right PPC increased the neural noise in this region (Miniussi *et al*., 2013) leading to a reduction in task performance. Along these lines, a study in the visual domain has shown impaired perceptual learning consolidation after anodal tDCS, which was interpreted in the context of a potential increase in noise and a decrease of the signal‐to‐noise ratio (Peters *et al*., 2013).

The results of our study therefore add to the growing evidence highlighting the diversity of effects that tDCS modulation can have on performance depending on the interaction between target site and task. They feed into a larger debate about the need to consider how baseline levels of cortical excitability within different neural systems contribute to task performance when attempting to develop optimal tDCS protocols (e.g. Krause & Cohen Kadosh, 2014). More studies including different stimulation intensities and durations, potentially in combination with brain imaging (e.g. Di Bernardi Luft *et al*., 2014), are needed to clarify the detailed effects of tDCS on cognitive performances.

The present study does not allow a distinction of the possible involvement of the right PPC for a specific stage of the memory process. It would be desirable to disentangle the different memory stages, encoding, retention and retrieval in a follow‐up study. Along these lines, a stage‐specific involvement of the left supramarginal gyrus during the retention and not encoding stage of pitch memory was revealed recently (Schaal *et al*., 2015). In that study, repetitive transcranial magnetic stimulation was applied during the retention stage or encoding phase of a pitch recognition task over the left supramarginal gyrus or vertex. The results showed increased reaction times selectively when stimulation was applied over the left supramarginal gyrus and during the retention period.

A further point to consider is the functional role that the right PPC plays in the melody memory process and to what extent the modulation effects can be explained according to this framework. The studies of Klostermann *et al*. (2009) and others associate the involvement of the PPCs with memory retrieval (for a review, see Rugg & Curran, 2007), which is also linked to attentional features in visual and non‐visual modalities (Shomstein & Yantis, 2006; for a review, see Ciaramelli *et al*., 2008). With respect to the results of the present study it is plausible that we modulated the attentional control of the right PPC and thereby created an imbalance of attentional awareness, which in turn led to a disruption of memory for the melodies. This disruption may have resulted from diverting attention from the melody memory task. Along these lines, Jacobson *et al*. (2012) showed that simultaneously applied anodal tDCS over the left superior parietal lobe and cathodal tDCS over the right inferior parietal lobe, compared with the opposite condition of stimulation, resulted in an attentional shift and significant facilitation of memory recognition for novel words. Furthermore, they also looked at familiarity and recollection processes but could only find a descriptive improvement for the recollection process in their paradigm and the difference did not reach significance. It is important to note that their recollection and familiarity analysis was based on confidence ratings, which is a slightly different approach compared with the remember/know paradigm used in the present study and could explain the different outcomes (Geraci *et al*., 2009).

In conclusion, the present study reveals a causal involvement of the right PPC for recognition memory of melodies by showing that anodal tDCS over the right PPC, compared with sham stimulation and anodal tDCS over the left PPC, leads to a decline in memory performance. Hereby, the effect may be based on the modulation of attentional control required for the melody recognition memory task. Furthermore, the deterioration can be traced back to the interference of anodal tDCS on conscious *remember* judgements, indicating that the right PPC is involved in the recollection process. It would also be desirable for future studies to investigate at which stage of the memory process, e.g. encoding or recognition, the right PPC is involved.
